# Conference Report: SECOND INDUSTRY WORKSHOP ON POLYMER COMPOSITE PROCESSING, Gaithersburg, MD May 18, 1990

**DOI:** 10.6028/jres.096.040

**Published:** 1991

**Authors:** S. S. Chang, D. L. Hunston

**Affiliations:** Polymers Division, National Institute of Standards and Technology, Gaithersburg, MD 20899

## 1. Introduction

The issue of international competitiveness has received much attention lately, and rightly so. One example is a recent Department of Commerce (DOC) study that was conducted to identify emerging technologies since they are critical tools for the development of better, more competitive products in the future. The results, which are detailed in a report [1] entitled “Emerging Technologies—A Survey of Technical and Economic Opportunities,” have identified Advanced Materials as one of the key technologies. This same conclusion was reached by a number of other industry and Government sponsored studies including those by the Aerospace Industries Association [2], “Key Technologies for the 1990s” and the Department of Defense [3], “Critical Technology Plan.” The Congress’s Office of Technology Assessment (OTA) reports $2 billion worth of advanced materials are currently replacing conventional materials annually [4] and predicts this will rise to $20 billion by the year 2000. The OTA study warns, however, that the emphasis on commercialization by Japan and Europe could put them in a very good position to compete for these markets. This warning is echoed by the DOC report which concludes that the United States is rapidly losing its lead in advanced materials.

The largest single item in the advanced material category is polymer based composites. The annual growth rate for polymer composites [5,6] is very high, 16%, but the continuation of this growth requires the expanded use of composites in mass-market, civilian applications. The major barrier to this is the high cost, and this is increasingly a concern for military and aerospace applications as well. A report [7] by Kline & Co. estimated that more than 70% of the cost for advanced composites is in the fabrication and, like a similar report [5] by Business Communications Co., concludes that improvements must be made if the potential of these materials is to be realized. The problem has arisen because the pressure for rapid implementation of composites has led the applications to outstrip the development of a corresponding science and technology base in fabrication. It is now generally recognized that a major effort is needed to correct this problem.

### 1.1 Purpose of Workshop

The field of composite fabrication is very complex with many potential areas to study, and thus, to be effective, the research activities must be focused on those aspects of the science base which will have the most direct impact on the development of cost effective processing. To identify these areas, an Industry Workshop on Polymer Composite Processing was held at the National Institute of Standards and Technology on October 7, 1987. The recommendations [8] from that meeting were used in planning a major expansion of the NIST composites program in 1988.

After more than 2 years, it was felt that the recommendations of the 1987 Workshop should be updated and refined so a second Industry Workshop was held at NIST on May 18, 1990. This Workshop also provided an opportunity to tell industry about the progress made by NIST’s composites research program since 1987. Of particular interest were the projects designed to address the recommendations of the 1987 meeting. A final objective of the Workshop was to seek industry’s advice and guidance for planning an expansion of the NIST effort. Such an expansion would address the scientific and technical questions associated with testing and prediction of performance properties in composites. To examine this area, the Workshop was asked to identify and discuss the performance issues that are most important to their industries. The results and discussions of the second Industry Workshop are summarized in this Report.

#### 1.1.1 Information Sought

To accomplish these goals, the attendees were asked to consider the time period from 5 to 15 years from now and answer three questions. First, what are the generic processing methods that will be of most interest to industry during this time period? Second, what are the scientific and technical barriers that hinder the implementation and effective use of these methods? Third, what are the performance issues that are most important for your industry?

#### 1.1.2 Workshop Composition

The meeting involved only industrial participants so that the results would reflect the position of industry. There were a total of 26 attendees representing 24 different company organizations. The attendees were asked to indicate which industry sectors they could represent and the breakdown was: 35% aerospace, 21% automotive, 15% electronics, 11% marine/hydrospace, 6% construction, and 12% other which includes prepreg fabrication, industrial applications, general part manufacturing, and database/design management. The attendees were split about evenly among users, suppliers, and those involved in both. The suppliers included manufacturers of resins and fibers as well as starting materials, such as preimpregnated fiber tape and cloth, and fabricators of small parts for the larger industrial users. A full attendance list was given elsewhere [9]. In addition, comments were supplied by scientists from two companies that could not participate in the Workshop but expressed great interest in the NIST research effort.

## 2. Workshop Program

The Workshop was a day long meeting whose agenda was given elsewhere [9]. It began with a review of the conclusions of the 1987 Workshop, and a brief overview of the NIST’s composites research program with emphasis on how it had responded to the 1987 recommendations. This was followed by presentations from representatives of four industry sectors: automotive, electronics, aerospace, and database/design. Each speaker reviewed the current relevance of the conclusions from the 1987 Workshop for their industries and suggested where revisions were needed for the 1990’s. Knowledgeable members of the audience augmented the presentation with comments based on their own experience and expertise.

After the industry presentations, a questionnaire was given to each attendee. This questionnaire, which is described below, provided an opportunity for each person to answer the questions posed in the Workshop and furnish other comments as well. While the attendees were completing this form, a more detailed look at the NIST research program was provided with presentations on six processing related projects. This occupied the remainder of the morning and early afternoon schedule. The questionnaires were collected before lunch and the evaluation begun at once so their results would be available for discussions in the afternoon.

During the last part of the meeting, Carl Johnson of Ford led discussions on processing methods, technical barriers, and performance issues. The preliminary results from the questionnaire were used to focus the deliberations. The goal was to reach a consensus among all industry sectors on answers to the three Workshop questions and other issues raised during the discussions. To a large extent, this was achieved although in a few cases, the answers were industry dependent. In addition, the issue of technologies that complement processing produced a large number of new ideas which made it difficult to finalize priorities during the discussions. As a result, a second questionnaire was sent to attendees by mail following the meeting so they could asssign priorities to the topics in this area. The Workshop was closed with a summary of the discussions and conclusions by Carl Johnson.

### 2.1 Review of 1987 Workshop

Industry representatives at the 1987 Workshop were asked to determine the processing methods that would be the most important in the future (5 to 15 years) and the scientific and technical barriers that prevent the optimal use of these methods today. The results are briefly outlined below and in Table 1.

#### 2.1.1 Processing Methods

The 1987 Workshop produced a consensus among the industry representatives on five processing methods. In order of decreasing priority they are: pressure molding, transfer molding, filament winding, thermoforming, and pultrusion. Pressure molding included both compression molding and autoclave processing. The first two methods were ranked about evenly and were rated significantly higher than the other three.

#### 2.1.2 Technologies that Complement Processing

In addition, two other technologies were identified as very important, but they can not really be classified as processing methods. Consequently, a new category was defined, namely technologies that complement processing. The first of two items in this list was alternate sources of energy input. This included heating by microwaves, lasers, hot gas jets, and similar techniques which have the potential for highly controlled energy input. The second technology was resin coating of fibers. Powder prepregging, commingled fibers, and similar methods for combining the two constituents in unique ways were included in this item.

#### 2.1.3 Scientific and Technical Barriers

In the area of scientific and technical barriers, the Workshop developed a list of six problem areas. Four of them involved the inability to understand and control various processing events: resin flow and fiber orientation (rated first), heat flow (rated second), morphology in partially crystalline systems and in multiphase toughened resins (rated fifth), and surface quality and dimensional stability (rated sixth). These areas are particularly important since they provide the targets for on-line process control which the Workshop regarded as the key to more rapid and reliable processing. The third most important area was fiber-matrix adhesion. It was felt that the measurement techniques for fiber-matrix adhesion needed to be improved while the factors which determine the bond strength must be better understood and controlled during processing. The problem area listed as fourth most important was data validation and test standardization. Of interest here were quality control tests, materials acceptance tests, and performance prediction tests.

#### 2.1.4 Material Systems with Potential for the Future

The final topic discussed in the 1987 Workshop was material systems. Although thermosetting resins were felt to be the most important at the present time, the attendees suggested three classes of materials that have great potential for the future and should be closely watched. These are thermoplastics, liquid crystal polymers, and molecular composites.

## 3. NIST Research Program

Only a brief summary of the NIST composite program will be presented here. Complete details on the program can be found in the Polymer Division’s Annual Report [10], The focus of the program is material science. The program generally uses existing materials, often model systems, and studies the changes that occur during processing. Processing invariably introduces microstructure which influences properties and so a second portion of the program concentrates on developing techniques to characterize this microstructure. Finally, the properties of the finished test piece are determined. The NIST program generally does not include synthesis of new materials although there are cooperative efforts with universities and industries where the co-participant performs the synthesis. At the other extreme, the performance of large structures is also outside the scope of the NIST effort which usually stops at the level of plates, tubes, or other very simple structures.

The two major program goals are: (1) to monitor, model, and ultimately control the chemical and physical changes that occur during processing in order to develop the tools needed for more rapid and reliable fabrication, and (2) to establish processing-microstructure-property relationships so improved performance and performance prediction can be achieved. As outlined in Table 2, the projects are divided into three tasks: Processing science, Microstructure characterization, and Laminate performance.

## 4. Result and Discussion of Second Workshop

Following the industry overviews, the first questionnaire was distributed, and while attendees filled it out, presentations were made on six NIST projects related to processing science. These presentations will not be described here, but are covered in the Polymer Division’s Annual Report [10]. The remainder of the meeting was then directed to a general discussion of the three questions the Workshop was asked to address. The results of the questionnaire formed the basis for the discussions.

### 4.1 Questionnaire

#### 4.1.1 First Questionnaire

The first questionnaire was divided into four sections. The initial page requested information on which industry sector/sectors the attendee could represent. The first section asked each respondent to identify and rank the most important scientific and technical barriers that hinder cost effective processing in their industry. The second section expanded each barrier by listing four to six subtopics so attendees could specify in more detail exactly where they saw the biggest challenges. The third section asked the respondent to identify and to assign priorities on the performance issues that are of most concern to their industry. The final section requested an update on the ranking of items in three categories: processing methods, important technologies that complement processing, and materials with potential for the future.

Each section of the questionnaire contained a list of possible answers based on the results of the 1987 Workshop and suggestions made by the attendees on their meeting registration forms. In addition, space was provided so last minute items could be added, and the attendees took advantage of the opportunity to include several important new topics, particularly in the area of technologies that complement processing.

#### 4.1.2 Second Questionnaire

The second questionnaire contained a list of all the suggestions for technologies that complement processing. It was mailed to each Workshop attendee, and they were asked to indicate their priorities and return the list for evaluation.

### 4.2 Questionnaire Analysis

The results from the questionnaire provide a good representation of the discussion and consensus in the Workshop. The questionnaires were evaluated with a point system. Each attendee was asked to rank in order of importance the answers to each question. Multiple answers could be given the same rank if they were equally important. The first place ranking for each question was given 4 points while the second place received 3 points, the third place 2 points, and the fourth place 1 point. The total points for each answer was then divided by the maximum points possible (i.e., if ranked number one by everybody) and multiplied by 100. This produced a scale which ranged from 100 to 0, where 0 indicated no one ranked the item in their top four. Since the questionnaire contained information on which industry sector the respondent represented, the results could be analyzed for differences between specific industries as well as general trends. The Workshop composition permitted examination of four industries: automotive, electronics, aerospace, and marine. Because the number of respondents in each sector is limited, the industry specific analysis is regarded as qualitative. Nevertheless, the results are quite informative.

#### 4.2.1 Processing Methods

The first issue addressed in the discussion was the selection of the most important processing method. A suggestion was made that the category of resin transfer molding be expanded to include related processes such as structural RIM (SRIM). Such fabrication methods are very closely related, share the same problems, and are appropriate to consider together. The term liquid molding was recommended as a more inclusive term. This change was made and is reflected in Table 3 which summarizes the results of the first questionnaire.

An analysis of the results by industry sector indicates that automotive listed liquid molding as most important method with pressure molding a second. Electronics listed press molding as the dominate area, but expressed interest in autoclave (pressure molding) and liquid molding as well. Aerospace listed autoclave as first but other methods, particularly liquid molding, were listed quite high. Marine also had a broad range of interests in all methods but listed pressure molding and liquid molding highest.

The general list of priorities is almost identical to that obtained at the last Workshop. Only two changes of any significance were noted. First, there was considerably more interest in fabrication by press under the category of pressure molding. Although led by the electronics industry, other sectors also expressed a stronger interest in this method than they did at the last Workshop. The second difference is the relative importance given to the top two ranked items, pressure molding and liquid molding, relative to the method ranked third. At the last Workshop pressure molding and liquid molding were clear winners, but now the advantage over the third place method is even greater than before. The high ranking given to these two methods reflects the fact that a broad range of industries considers them very important. The increased interest in press molding mentioned above is one example. Moreover, both the aerospace and marine industries expressed interest in a broader range of methods than was the case 2 years ago and liquid molding and press molding receiving much of the increased attention. In the last survey aerospace ranked transfer molding as 10th and liquid molding was not listed at all. This time liquid molding ranked a close second. The potential cost advantages of these methods obviously pays a major role here.

#### 4.2.2 Scientific and Technical Barriers

The second item discussed were the scientific and technical barriers to utilization of improved processing methods. The results of questionnaire one are shown in Table 4.

An analysis of the results by industry indicates that aerospace rated the first four topics in this order while electronics rated the first two in this order but lists fiber-matrix adhesion, morphology, and surface quality/dimensional tolerance as tied for third. Automotive listed resin flow/fiber orientation as one, surface quality/dimensional tolerance as two, data validation/test standards as three and process monitoring and control as four. Marine listed process monitoring and control as one, fiber-matrix adhesion as two and resin flow/fiber orientation as three.

The results here were very similar to those in the last Workshop including the interest of automotive and electronics in surface quality and dimensional tolerance. There were, however, two important changes. First, heat flow and temperature gradients fell from number two to number seven. One possible explanation for this is that improvements in modeling capabilities have made the prediction of heat flow more accurate and reliable than it was 2 years ago. The second major change was the inclusion of process monitoring and control as a separate item rather than have it included implicitly as was done last time. The reason for this was that the technology had developed to the place where it is now useful to address this topic directly. When listed in this way, every industry sector rated it as either first or second in importance.

An important point mentioned several times during the discussion was the trend toward more complex parts. Current production often involves fabrication of large, three-dimensional components. In addition, there is much interest in thick section composites (25 cm or more) for a number of applications. This increases the need to address the barriers above both because the processing is more complex and the costs associated with failure are far greater.

After an overall ranking of the barriers, the questionnaire asked attendees to explore the importance of specific topics related to each barrier. The results of detailed analysis of these barriers from the second questionnaire and associated discussions during the meeting were given elsewhere [9].

#### 4.2.3 Technologies that Complement Processing

The Workshop discussed the technologies that complement processing and identified a number of areas that had not been mentioned either at the previous Workshop or in suggestions offered on the meeting registration forms. Consequently, a second questionnaire which included these new technologies was developed and completed by mail. For several of the technologies, a number of specific topics were listed, and attendees were invited to indicate if they considered any of these topics to be particularly important. The results of the questionnaire are given in Table 5. For those cases where a number of attendees indicated a high degree of interest in a specific topic for a technology, these topics are also included in Table 5.

The highest ranked technology is fiber placement. This refers to an advancement on filament winding in which a number of toes are applied simultaneously. Each toe has its own pressure roller that positions and attaches it to the part using either tack (thermosets) or on-line consolidation (thermoplastics). This makes it possible to do complex shapes with concave regions and other desirable features. The individual toes can be cut and stopped or restarted when desired during processing. The result is a very versatile and rapid fabrication process. The second technology is alternate forms of prepreg preparation. This includes a number of new technologies, but the attendees selected powder prepregging and commingled fibers as particularly important. Joining is the technology rated third. Joining can mean thermoplastic welding, adhesive bonding, mechanical fasteners, etc. A number of attendees singled out adhesive bonding as particularly important for the future. The technology ranked fourth was preform preparation which includes trimming, stitching, braiding, etc., as well as automation of these processes. This technology was followed by recycling, environmental safety, and tooling in the list of priorities. The final technology listed was alternate sources of energy. A number of examples were discussed during the Workshop, but the responses to the questionnaire gave special attention to microwave radiation and heat assisted fiber placement.

Relative to the results in the 1987 Workshop, the major difference is the increased number of technologies with high interest. Only two topics, prepreg preparation and alternate sources of energy, were chosen in 1987. A detailed analysis for specific industry sectors indicates that the top four aerospace priorities were in agreement with this list. The highest rated automotive items differ in that preform fabrication was listed number one and recycling was rated number four instead of prepreg preparation. Electronics ranked prepreg preparation, joining, and alternate sources of energy as top priorities. In the list for marine, preform preparation was not ranked highly while fiber placement, environmental safety and tooling were.

The high ranking given to preform fabrication by the automotive sector was expected although some attendees from this sector rated it rather low. Perhaps, this reflects the difference between those interested in primary structure applications and those involved in sheet molding compound for body panels. A high rating was given to recycling by both automotive and marine, but this was counterbalanced by aerospace where everyone rated this topic quite low. This is understandable in light of the production volumes involved for the different industries.

#### 4.2.4 Materials with Potential for the Future

The Workshop also discussed material systems that have potential for the future and therefore should be watched closely. Everyone agreed that thermosets are very important today and will continue to be widely used in the future. Some attendees felt that thermoplastics (TPs) were also viable candidate materials at present, while others believe the cost effectiveness of TPs is sill unproven. There was general agreement, however, that TPs had great potential. Both amorphous and partially crystalline TPs were considered and no distinction was made during the discussions.

In addition to TPs, four other material systems were identified as having great potential. All five are listed in Table 6. The first three were included in the initial questionnaire and the ranking by attendees was equally distributed among them. During the discussions, two additional items were added. These items were also considered as very important so no effort was made to prioritize this list.

The term smart materials was used to designate a variety of material systems which are either active, i.e., piezoelectric, pyroelectric, etc., or contain built-in sensors, i.e., fiber optics, piezoelectric layers, etc. The category of specialized polymer systems includes blends, interpenetrating networks, cyclic oligomers, etc. Such systems have the potential for significantly improved properties relative to simple polymers. The area of molecular composites was also discussed, and it was concluded that they have much potential but cost and processing difficulties present barriers to their use. Finally, liquid crystal polymers were considered, and there was much excitement about their potential to build-in specific properties, i.e., anisotropy generated by controlled molding. The ability to obtain excellent properties in one direction, however, can be compromised if the properties on other directions are poor. A better understanding of these materials and their processing was viewed as the key to realizing their potential.

#### 4.2.5 Performance Properties

The final issue addressed by the Workshop was performance properties. The list of possible problem areas included on the questionnaire began with the four topics identified by the Automotive Composites Consortium and then added items known to be of interest to aerospace and electronics as well as topics suggested by attendees on their registration forms. Table 7 shows the results from the questionnaire for the seven topics rated as most important.

A detailed analysis by industry sector shows that automotive listed impact and environmental effects as one and two while thermal stability was third and dimensional stability forth. Electronics listed environmental effects first with impact and dimensional stability tied for second. Thermal stability tied delamination for fourth. Aerospace listed delamination as first while electronics listed it as fourth. This is the main reason delamination appears so high since others ranked it quite low. Aerospace listed impact as second, environmental effects as third, and dimensional and thermal stability as tied for fourth. Marine listed fatigue and impact as first with creep and environmental effects tied for third.

The results showed impact (which probably includes crash worthiness for automotive) and environmental effects as high priorities for all industry sectors. Beyond that the need depended on the industry. In aerospace the overriding concern was delamination; in electronics it was dimensional stability, and in marine, it was fatigue. There was also concern in a number of industries about creep, particularly for applications using thermoplastics, and thermal stability in applications where dimensional stability (thermal expansion) or high temperature are important. The discussion suggested, however, that these differences between industries may become less important as time passes. For example, the reason fatigue was not ranked higher in automotive and aerospace is that the designs are now dominated by crash worthiness and delamination. Once these problems are solved, fatigue may become an important concern. The discussion also emphasized the importance of processing in determining the microstructure that controls performance. The lack of understanding in this area was considered an important problem.

## 5. Conclusions

Two processing methods were selected as by far the most important fabrication techniques for the future: pressure molding and liquid molding. Pressure molding was defined to include flat bed press molding, compression molding, and autoclave molding. The term liquid molding was used to describe resin transfer molding (RTM) and structural reaction injection molding (high speed RTM).

The marine industry expressed a broad range of interests, while automotive’s primary focus is liquid molding, aerospace’s is autoclave molding, and electronic’s is press molding. Surprisingly, however, all industry sectors expressed interest in a variety of pressure and liquid molding techniques.

Three additional processing methods were identified as being important for the future: filament winding, thermoforming, and pultrusion.

Seven scientific and technical barriers to the full exploitation of the processing methods outlined above were identified. The highest priority was the need to understand and control resin flow and fiber orientation. The importance of resin flow was associated with the problems of void formation, mold filling, and edge and corner flows. In connection with fibers, the major concerns were fiber wetting, fiber alignment, and orientation control.

The second highest priority barrier was the need for process monitoring sensors for on-line process control. This included the development of new techniques and the improvement of existing methods. Current sensors and electronics need to be made more rugged to operate effectively on the factory floor, and the output of the sensors must be better understood and more closely linked to process control.

The third highest ranked barrier was the need to understand and control the fiber-matrix interface. Of particular concern is the area of test methods where current techniques are difficult to use and interpret, or not developed to the point where clear correlations with composite properties exists. Another area where it was felt that more understanding was needed was surface treatment.

Data validation and testing standards was another area that needed more study. Measurements related to performance were a particular concern, but quality control testing was also of great interest. Measurement of viscosity and degree of cure were particularly important here since the focus was processing.

The inability to determine the optimum morphology and achieve it during processing was another area of great concern. All industry sectors felt this was important since morphology often plays a significant role in toughness. The aerospace and automotive industries also expressed a concern about the control of crystallinity in partially crystalline thermoplastics.

The sixth most important barrier was the inability to adequately control surface quality and dimensional stability. Although all industry sectors had concerns in this area, automotive and electronics rated this area as second and tied for third respectively in their priority lists.

The final barrier selected was heat flow. This area was second on the priority list generated at the 1987 Workshop. This change may be due to the improvements that have occurred during the past several years that now make modelling of heat flow easier and more accurate.

The Workshop also identified and assigned priorities to eight technologies that complement processing and are important for the future. The two highest ranked items were fiber placement and new methods to prepare prepreg, i.e., powder prepregging, commingled fibers, etc. Joining was listed as third, and although both adhesive bonding and mechanical fasteners were mentioned, the former received by far the most attention. Preform preparation was listed next primarily on the strength of the number one ranking given by automotive. Recycling, environmental safety and tooling were listed next in that order. As might be expected, the greatest interest in these topics was for mass production markets, i.e., automotive and marine. Alternate sources of energy, which includes microwave heating, heat assisted fiber placement, etc., was ranked eighth but had several strong supporters in electronics and marine.

The majority of people at the Workshop felt that thermosets were still the dominant resin system in their applications. A number of attendees, however, were actively engaged in thermoplastic development and everyone felt these materials had great potential for the future if their cost effectiveness could be established. Thermoplastics were therefore classified as a material to be watched closely for future development. Four other classes of materials were also included on this list: liquid crystal polymers, molecular composites, smart materials, and specialized polymer system. Smart materials include systems with either built-in sensors or active components such as piezoelectric layers. Specialized systems include blends, interpenetrating networks, cyclic oligomers, etc. The last two categories were not on the this list at the Workshop 3 years ago and represent new technologies. In addition, the interest in liquid crystal polymers was somewhat greater than it was 3 years ago.

The Workshop also selected seven performance issues that they felt were critical to the future use of composite materials. In order of priority they are: impact, environmental attack, delamination, dimensional changes, thermal stability, fatigue, and creep. For the highest ranked topics there was a surprising consensus with all industry sectors ranking impact and environmental attack in their top three items. For automotive impact included crash worthiness. Beyond this point, the priorities differed for each industry. Aerospace listed delamination as their highest priority and dimensional changes as fourth. Automotive added thermal stability and dimensional changes as third and forth. Electronics was similar to automotive with slight differences in ordering. Marine listed fatigue and impact as first with creep and environmental attack as tied for third. The differences generally reflect one or two overriding concerns for the particular application, for example, delamination in aerospace. As these concerns are successfully addressed, other problem areas will become more important, i.e., fatigue will become more of a concern to aerospace if and when the delamination problem is solved.

## Figures and Tables

**Table 1:** Conclusion of 1987 workshop

Most important processing methods (rank)
Pressure molding (1)
Transfer molding (2)
Filament winding (3)
Thermoforming (4)
Pultrusion (5)
Important technologies that complement processing
Alternate sources of energy
Resin coating of fibers: Preparation and processing
Most important scientific and technical barriers (rank)
Inability to understand and control
Resin flow—fiber orientation (1)
Heat flow (2)
Morphology (5)
Surface quality—dimensional Tolerances (6)
Fiber-matrix adhesion (3)
Data validation—test standardization (4)
Potentially important materials for the future
Thermoplastics
Liquid crystalline polymers
Molecular composites

**Table 2:** NIST Composite Research Program

Processing science
Process monitoring—10 monitoring techniques
Processing facilities
Resin transfer molding (RTM)
Automated press
Autoclave/prepregger
Process modelling—RTM
Computation
Flow visualization
Process control
Microstructure characterization
Resin
Thermoset—network structure
Undeformed state
Deformed state
Thermoplastic—crystallinity
Fiber
Gel spun fiber—gel structure
Interface
Structure of glass/resin interface
Composite—thick section
Through thickness variations
Laminate performance
Test methods
Delamination
Fiber-matrix interface strength
Modelling
Delamination
Buckling in compression
Failure mechanisms —resin, adhesive, composite
Crack-tip visualization
Toughening
Physical aging

**Table 3:** Processing methods

Method	Score
Pressure molding	84
Liquid molding	82
Filament winding	39
Thermoforming	29
Pultrusion	21

**Table 4:** Processing barriers

Barriers	Score
Resin flow / fiber orientation	69
Process monitoring and control	52
Fiber-matrix interface	44
Data validation / test standards	33
Morphology understanding and control	28
Surface quality/dimensional tolerance	23
Heat flow	21

**Table 5:** Technologies that complement processing

Technology	Score
Fiber placement	53
Prepreg preparation	47
Powder prepregging	
Commingled fibers	
Joining	40
Adhesive bonding	
Preform preparation	33
Recycling	23
Environmental safety	21
Tooling	21
Alternate sources of energy	17
Microwave	
Heat assisted fiber placement	

**Table 6:** Materials with potential for the future

Liquid crystal polymers
Thermoplastic polymers
Molecular composites
Smart materials
Specialized polymer systems

**Table 7:** Performance properties

Property	Score
Impact	61
Environmental effects	57
Delamination	43
Dimensional changes	43
Thermal stability	31
Fatigue	27
Creep	21
